# Acute Renal Shutdown Following Femoral Central Venous Catheterization: Iatrogenic or Spontaneous Rupture of the Inferior Epigastric Artery

**DOI:** 10.7759/cureus.73038

**Published:** 2024-11-05

**Authors:** Rajashekar R Mudaraddi, Mohan Babu A, Hany Fawzi Greiss, Osama Sami Maki Al Ani

**Affiliations:** 1 Anesthesiology, Rashid Hospital, Dubai, ARE; 2 Anesthesiology and Critical Care, Rashid Hospital, Dubai, ARE

**Keywords:** computerized tomography, femoral central, inferior epigastric artery, stent graft, ultrasound-guided

## Abstract

Femoral central venous catheterization is a commonly performed procedure in the intensive care unit or ward in a hospital setting. Unfortunately, the venous catheterization procedure can cause insertion-related complications, commonly referred to as mechanical complications, which can range from being clinically insignificant to life-threatening if untreated. The femoral vein is chosen due to its low risk for complications such as bleeding, which are easy to control when the patient is on anticoagulants. Although the infection rate is high with femoral central line insertion, we prefer it over others given the history of anticoagulants. Here, we present a case of acute renal shutdown following the femoral central line given Inferior epigastric artery injury with hematoma compressing the urinary bladder. The early recognition of complications led to successful management of the patient.

## Introduction

Central line insertion is one of the common procedures performed in hospital settings to administer medication to patients [1]. Femoral venous cannulation is performed in the emergency department every day for various reasons. One such use is for venous access when peripheral access is unobtainable or not appropriate [1-3]. The use of ultrasound for the insertion has led to fewer complications [4,5]. The complications depend upon patient factors, operator factors, and clinical scenarios. Here, we describe an unusual case of femoral central venous catheter-related complication. Although many mechanical complications have been published in the literature, the case presented here posed a diagnostic dilemma to the clinicians treating the patient. Identification of the complication and timely intervention saved the patient from life-threatening complications.

## Case presentation

**Figure 1:**
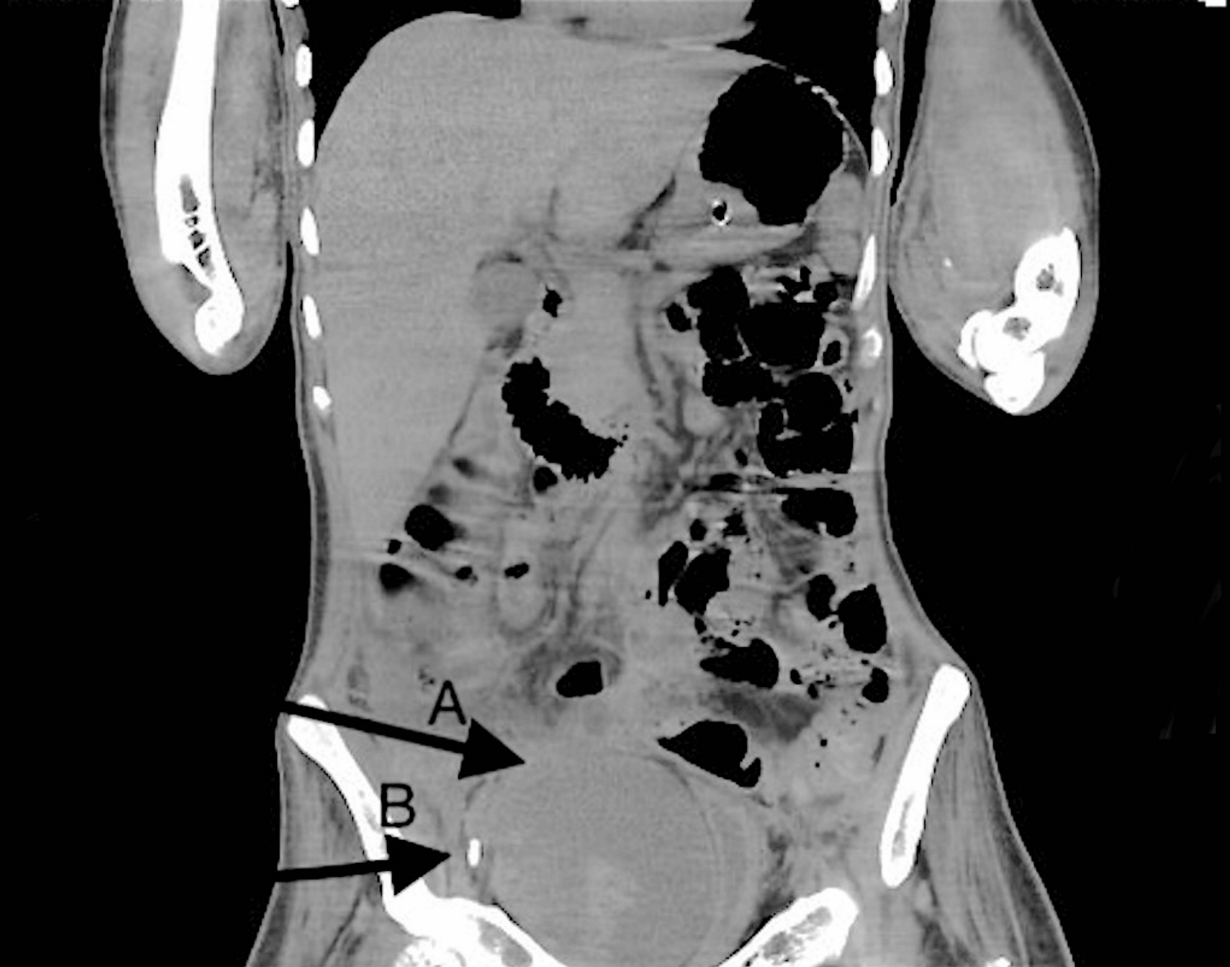
Hematoma compressing the bladder. (A) Hematoma compressing the bladder. (B) Femoral central line.

A 33-year-old male patient with a history of polytrauma including cervical spine fracture with quadriplegia and prolonged hospital stay was referred to us for difficult peripheral Intravenous access. The patient was operated on for anterior cervical disc fusion from C4 to C6. Postoperatively, the patient was on a ventilator, eventually tracheostomized, and was on supportive care after weaning from the ventilator. The patient had a history of pulmonary embolism in the hospital course and was on therapeutic low-molecular-weight heparin 80 mg two times a day. He had contractures in all limbs. The previous laboratory investigations were normal. The patient was attended to by our team. An initial attempt of right femoral central line insertion with ultrasound failed. Although an arterial puncture occurred in the first two attempts, later insertion was successful. After four hours of insertion, the patient developed abdominal distension on the right side with reduced urine output. There was initially oliguria followed by anuria. The serum creatinine was 0.3 mg/dL but raised to 2.6 mg/dL, with the glomerular filtration rate falling from 161 mL/minute to 32 mL/minute. Hemoglobin dropped from 11 g/dL to 7 g /dL. There was tachycardia with maintained blood pressure. We suspected possible hematoma due to iatrogenic injury or spontaneous bleeding given the therapeutic administration of low-molecular-weight heparin. Emergency CT of the abdomen and pelvis showed a large pelvic complex heterogeneous collection extending into the right rectus sheath, likely representing a large hematoma with bladder compression and hydroureteronephrosis (Figure 1).

**Figure 2:**
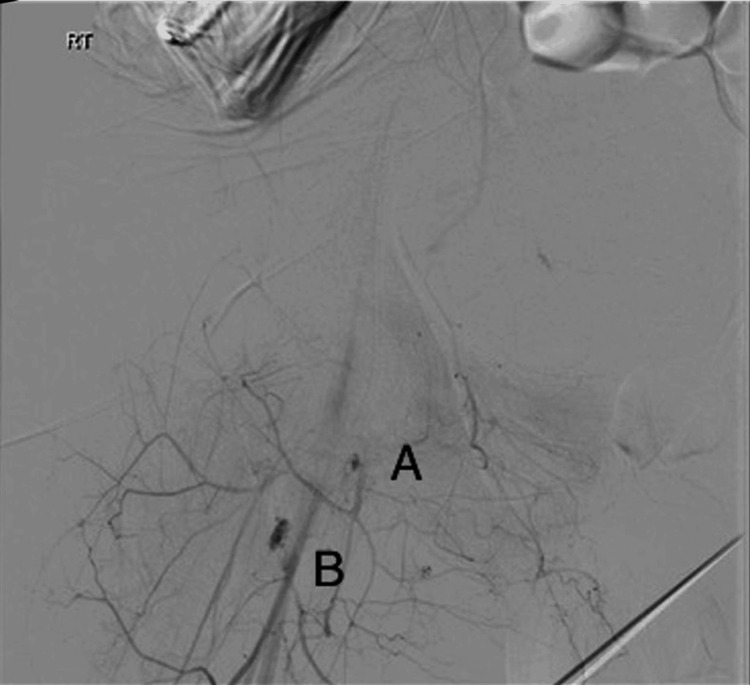
Bleeding points on angiography. (A) Bleeding from the inferior epigastric artery. (B) Bleeding from the superficial femoral artery.

The decision was made to find the source of the bleeding. The patient was shifted to the Angio suite and underwent a CT angiogram which showed active bleeding in the right inferior epigastric artery and the proximal third of the right superficial femoral artery (Figure 2).

**Figure 3:**
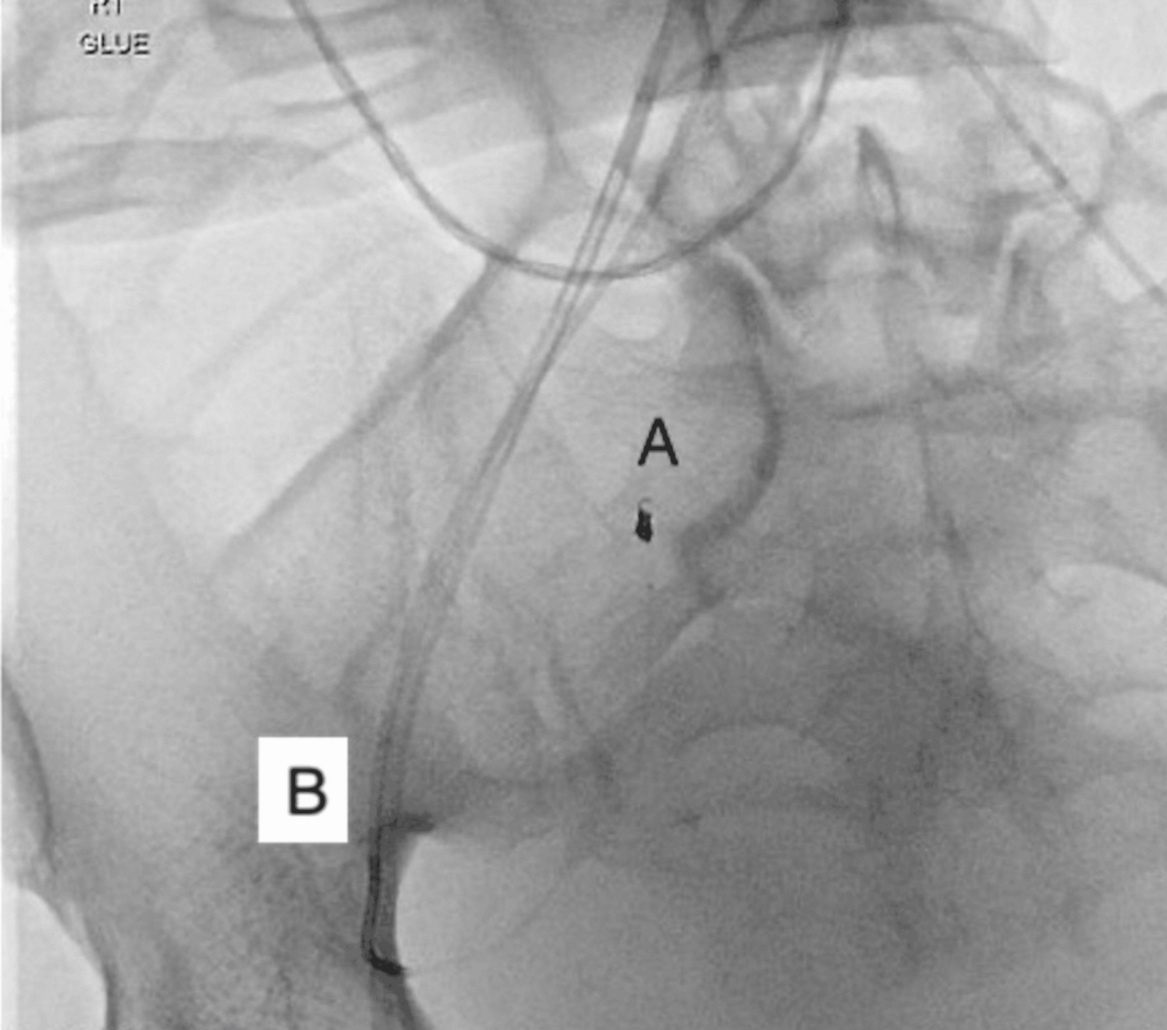
Coiling of the inferior epigastric artery with stent graft to the femoral artery. (A) Coiling of the inferior epigastric artery. (B) Stent graft to the superficial femoral artery.

Selective embolization of the right inferior epigastric artery was done using coil and glue. A covered stent was deployed at the proximal third of the right superficial femoral artery (Figure 3) under monitored anesthesia care. A control angiogram showed no active bleeding. Early detection of the complication and timely intervention saved the patient from further complications.

## Discussion

Although often necessary and beneficial, femoral central venous cannulation (CVC) is associated with significant risks that include arterial puncture, hematoma formation, guidewire loss, line malposition, and infection [1-3]. Direct ultrasound visualization of the needle tip and guidewire entering the vessel can reduce these complications [4].

Clinicians might prefer the femoral location for CVC as opposed to the internal jugular or subclavian veins in several clinical situations such as cardiac or respiratory arrest. Femoral veins can offer easier access and free the chest for compressions [1]. Even in a patient with coagulopathy, the femoral line is preferred given easy compression to control the bleeding [1]. Despite the ease of access, a femoral central line is not without complications, as described in our case.

Cases of femoral central venous complications have been reported [5]. The use of real-time ultrasound guidance is strongly recommended for central venous access, as it increases success and reduces the number of mechanical complications [4,5]. General barriers to ultrasound-guided CVC include access to equipment, inadequate amount of time, and lack of proper training. Although training individuals for ultrasound-guided CVC insertion, complications still occur, with patient factors and operator factors playing a crucial role [1]. Complications of femoral vein cannulation include puncture-site infection, local hemorrhage from the femoral artery or vein, femoral vein thrombosis, phlebitis, and arteriovenous fistula [1-6]. Retroperitoneal hemorrhage (RPH) is the most serious complication of femoral vein catheterization, and the incidence of RPH was estimated to be around 0.5% [5]. In a previously reported case, femoral CVC entering the iliolumbar vein was discovered during bone scintigraphy [3]. In our case, the patient was bed-bound with difficult anatomy because of spasticity in the lower limb. The patient was also on therapeutic low-molecular-weight heparin. The cause of bleeding appeared iatrogenic but spontaneous bleeding cannot be ruled out because of anticoagulants. The timely diagnosis of expected complications and availability of resources helped determine the cause of acute renal shutdown and treatment.

## Conclusions

Although femoral central venous catheterization is a safe procedure with the use of ultrasound, complications are reported. Early recognition and intervention help save patients from life-threatening complications. The complications should be identified as patient safety is a priority after any procedure. Regular follow-up of patients can help detect unanticipated rare complications.
